# A Re-Annotation of the *Saccharomyces Cerevisiae* Genome

**DOI:** 10.1002/cfg.86

**Published:** 2001-06

**Authors:** V. Wood, K. M. Rutherford, A Ivens, M-A Rajandream, B. Barrell

**Affiliations:** The Sanger Centre Wellcome Trust Genome Campus Hinxton, Cambridge CB10 1SA UK

## Abstract

Discrepancies in gene and orphan number indicated by previous analyses suggest that *S. cerevisiae* would benefit from a consistent re-annotation. In this analysis three new genes are identified and 46 alterations to gene coordinates are described. 370 ORFs are defined as totally spurious ORFs which should be disregarded. At least a further 193 genes could be described as very hypothetical, based on a number of criteria. It was found that disparate genes with sequence overlaps over ten amino acids (especially at the N-terminus) are rare in both S. *cerevisiae* and *Sz.* pombe. A new S. *cerevisiae* gene number estimate with an upper limit of 5804 is proposed, but after the removal of very hypothetical genes and pseudogenes this is reduced to 5570. Although this is likely to be closer to the true upper limit, it is still predicted to be an overestimate of gene number. A complete list of revised gene coordinates is available from the Sanger Centre (*S. cerevisiae* reannotation: ftp://ftp/pub/yeast/SCreannotation).


**Comparative and Functional Genomics**
*Comp Funct Genom* 2001; **2**: 143–154.
**DOI:** 10.1002/cfg.86

Research Article

### A Re-annotation of the *Saccharomyces cerevisiae* Genome

V. Wood*, K. M. Rutherford, A Ivens, M-A Rajandream and B. Barrell
*The Sanger Centre, Wellcome Trust Genome Campus, Hinxton, Cambridge CB10 1SA, UK*

*Correspondence to:
V. Wood, The Sanger Centre, Wellcome Trust Genome Campus, Hinxton, Cambridge CB10 1SA, UK.
E-mail: val@sanger.ac.uk

Received: 14 March 2001
Accepted: 19 April 2001

### Abstract

**Discrepancies in gene and orphan number indicated by previous analyses suggest that *S. cerevisiae* would benefit from a consistent re-annotation. In this analysis three new genes are identified and 46 alterations to gene coordinates are described. 370 ORFs are defined as totally spurious ORFs which should be disregarded. At least a further 193 genes could be described as very hypothetical, based on a number of criteria.**

**It was found that disparate genes with sequence overlaps over ten amino acids (especially at the N-terminus) are rare in both *S. cerevisiae* and *Sz. pombe*. A new *S. cerevisiae* gene number estimate with an upper limit of 5804 is proposed, but after the removal of very hypothetical genes and pseudogenes this is reduced to 5570. Although this is likely to be closer to the true upper limit, it is still predicted to be an overestimate of gene number. A complete list of revised gene coordinates is available from the Sanger Centre (*S. cerevisiae* reannotation: ftp://ftp/pub/yeast/SCreannotation).** Copyright © 2001 John Wiley & Sons, Ltd.

**Keywords: annotation; *Schizosaccharomyces pombe*; *Sacccharomyces cerevisiae*; comparative genomics; sequence orphans; hypothetical proteins**

### Introduction

### Background

The publication in 1996 of the first complete eukaryotic genome sequence, that of *Saccharomyces cerevisiae*, heralded a new era in biology (Goffeau *et al.*, 1996). This resource not only benefited those investigating *S. cerevisiae*, but also enabled inferences from the functional data to be transferred to a diverse range of other organisms. Unexpectedly, a significant proportion (56%) of annotated genes had not been studied previously, despite more than 50 years of traditional biochemistry and genetics (Oliver *et al.*, 1992, Oliver, 1996, Mewes *et al.*, 1997). This observation stimulated the application of functional genomics technologies to characterise these genes and their products, either gene-by-gene in small laboratories, or on a larger scale in some research institutes (Hieter and Boguski, 1997).

In the five years since the *S. cerevisiae* genome was sequenced, the majority (70%) of the predicted genes have been assigned an initial functional characterisation in the Yeast Protein Database (YPD, Proteome Inc. **http://www.proteome.com/databases/index.html**). Establishing the functional inter-relationships between all the genes in a genome requires, in the first instance, the assignment of genes to preliminary functional classes. These initial assignments will authenticate predicted genes as coding entities and partition the data into categories for subsequent biological analyses. However, it will be difficult to assess when this milestone has been reached since the exact number of genes in *S. cerevisiae* is still unclear: the Munich Information Center for Protein Sequences (MIPS) database has a protein complement of 6368 (**http://www.mips.biochem.mpg.de/proj/yeast**) Saccharomyces Genome Database (SGD) has 6310 (**http://genome-www.stanford.edu/Saccharomyces/**), and YPD has 6142 as of 26 January 2001.

It is likely that a significant cause of the discrepancies between these gene numbers are due to small, fortuitously occurring ORFs (open reading frames), which are notoriously difficult to

Copyright © 2001 John Wiley & Sons, Ltd.

distinguish from real genes (Dujon, 1996). In the original *S. cerevisiae* annotation, only ORFs greater than 100 amino acids in size were considered. This threshold was imposed in order to reduce the chance of missing small proteins without over-prediction due to the statistically expected frequency of small ORFs (Sharp and Cowe, 1991). Those without assigned function or homologues were designated *sequence orphans* (Dujon, 1996). As genome sequencing proceeded, the ratio of orphans to ORFs with homologues increased rapidly- so much so that this phenomenon was termed '*The mystery of orphans*' (Dujon, 1996).

The existence of relatively high numbers of orphans can only be attributed to one or a combination of the following:

1. They may simply be spurious ORFs. In *S. cerevisiae*, a number of predicted genes are also completely or substantially overlapping with defined coding features and should therefore be *disregarded*.
2. They may arise due to the acquisition of novel species-specific functions.
3. They escape functional characterisation by homology because they are rapidly evolving.
4. Identifiable homologues in other organisms exist, but these have not yet been sequenced.

The question of *S. cerevisiae* gene number has been addressed many times, with differing outcomes. Mackiewicz *et al.* (1999) estimated the total number of protein coding ORFs to be 4800, based on their sequence properties. Zhang and Wang (2000) calculated the likely number to be $\leq 5645$, based on the assumption that unknown genes have similar statistical properties to known genes. As part of the Genolevures project, Blandin *et al.* (2000) performed a consistent re-annotation of the *S. cerevisiae* genome using uniform criteria, revealing 50 possible novel genes and 26 gene extensions. They proposed a protein coding gene set of *at least* 5600 genes. As part of the same initiative, Malpertuy *et al.* (2000) estimate that the *S. cerevisiae* genome contains 5651 actual protein coding genes (including the 50 new predictions), and that the public databases contain 612 predicted ORFs that are not protein coding.

The availability of an additional yeast genome, that of *Schizosaccharomyces pombe* (fission yeast), which has 99.5% of its coding sequence annotated and deposited in the EMBL database (manuscript in preparation), will allow the comparison of the complete genomes and proteomes of two well-studied unicellular eukaryotes, which diverged around 330 million years ago (Berbee and Taylor, 1993).

### Aims

The discrepancies in gene and orphan numbers proposed by previous analyses suggested that *S. cerevisiae* would benefit from a consistent reannotation, applying new analytical methods and incorporating the data which have become available over the last four years. In doing so, we wished to achieve:

1. The refinement of gene complement.
2. The classification of orphans into hypothetical, very hypothetical, and spurious ORFs which should be disregarded.
3. The identification of gene prediction errors.
4. The identification of new genes.

The *Sz. pombe* genome annotation effort has benefited immensely from the availability of the complete genome of *S. cerevisiae*. The analysis methods used for the *Sz. pombe* genome combine *ab initio* gene prediction algorithms and homology search results with rigorous manual inspection of biological context (Xiang *et al.*, 2000). In addition, consistency checks using available cDNAs and ESTs have been routinely performed, and new experimental data from the fission yeast community immediately incorporated into the dataset. We believe that these methods provide an accurate, detailed gene set for this organism. The *Sz. pombe* analysis procedure has been applied to *S. cerevisiae* in order to define an up-to-date non-redundant gene set with consistent annotations and a new estimate of orphan numbers.

### Methods

### DNA sequences

The sequences of the 16 *S. cerevisiae* chromosomes, and associated ORF translations, were downloaded from SGD on the 16th November 2000. ORF coordinates were then converted into EMBL feature table format and imported into the Artemis sequence analysis and annotation tool (Rutherford *et al.*, 2000).

Copyright © 2001 John Wiley & Sons, Ltd. 

### Analysis procedure

A number of standard analysis tools were used to assist the interpretation of the sequence data (as applied to the *Sz. pombe* genome) (Xiang *et al.*, 2000). Searches were performed against public databases (SWISS-PROT and TrEMBL (Bairoch and Apweiler, 1999), EMBL (Stoesser *et al.*, 1999), Pfam (Bateman *et al.*, 1999), and PROSITE (Bairoch, 1994)) using standard software (BLAST (Altschul *et al.*, 1990), MSPcrunch (Sonnhammer and Durbin, 1994), tRNAscan (Lowe and Eddy, 1997), FASTA (Pearson and Lipman, 1988) and Genewise (Birney *et al.*, 1996)), to complete a series of automated analyses. This enabled annotated DNA and protein features to be confirmed. Other elements not included in the SGD annotation (experimentally identified snoRNAs and other cellular RNAs, omitted LTRs, and protein domains), were also mapped onto the sequence using in-house Perl scripts. *De novo* gene predictions were not performed as part of this analysis.

### New genes

In the *Sz. pombe* genome, more than 300 genes have been identified which are conserved at the protein level in other organisms, but absent from the *S. cerevisiae* dataset (manuscript in preparation). Some of these were small genes (70–150 amino acids); TBLASTN searches were conducted to determine whether these small genes had been omitted from the initial *S. cerevisiae* gene predictions.

### New gene coordinates

Within the annotation tool Artemis (Rutherford *et al.*, 2000), FASTA alignments were performed on existing gene predictions, to assess their accuracy. Overlapping ORFs were subject to systematic manual inspection to determine whether the correction of frameshifts or sequencing errors could extend homology, by merging existing genes or increasing their length.

### Disregarded spurious ORFs, overlapping with real genes

ORFs which have all, or the majority of their translation overlapping with other annotated features, were individually assessed for similarity to all organisms, as described in *New gene coordinates* above, together with experimental data if available. For ORFs to be considered as spurious, they had to meet all of the following criteria:

1. Small size (35–250 amino acids).
2. Absence of similarity to known proteins.
3. Absence of functional data which could not have been generated by the real overlapping gene.
4. Greater than 25% overlap at the N-terminus or 50% overlap at the C terminus with another coding feature; overlap with another feature at both ends; or ORF containing a tRNA.

Transposon fragments were also removed.

### Very hypothetical ORFs

In *Sz. pombe*, 177 ORFs which are considered unlikely to be coding but cannot yet be dismissed as spurious have been assigned as *very hypothetical* according to the following criteria:

1. Small size ( 100–250 amino acids).
2. Absence of similarity to other known proteins.
3. Overlap with other features, particularly at the N-terminus, where they might interfere with promoters (the overlaps in these cases are smaller than those observed in disregarded ORFs).
4. Extreme GC content.

The annotation of *Sz. pombe* adequately discriminates between *very hypothetical proteins* and real genes and this approach has been applied to a re-annotation of the *S. cerevisiae* genome.

### Results

### New genes identified

Three new genes were identified; 1. *YBL071W-a* a hypothetical conserved protein (simultaneously identified by Blandin *et al.*) 2. *YAL044W-a*, the homologue of *Sz. pombe uvi31* 3. *YDL085C-a*, the homologue of the human *4F5S* disease-associated gene. The new genes and coordinates are listed in Table 1.

### New coordinates (merged or extended genes)

The complete list of 46 proposed alterations to gene coordinates are presented in Table 1. Some of these changes have already been confirmed experimentally and deposited in the SWISS-PROT database

Copyright © 2001 John Wiley & Sons, Ltd. 

Table 1. New, merged or extended genes

| Chr. | CDS | Comment | NewCoordinates |
|---|---|---|---|
| I | YAL044W-a | New gene | 57520..57852 |
| II | YBL071W-a | New gene | 89973..90221 |
| IV | YDL085C-a | New gene | 302463..302669 |
| I | YAL065C+YAL064C-A | 1 | 11566..13173,13177..13362,13367..13744 |
| I | YAR066W+YAR068W | 1 | 221035..221643,221647..222930 |
| I | IMD1 | 1or2 | 227728..228844,228846..229303 |
| II | VMA2 | extended | 491228..492781 |
| III | YCR099C+YCR100C+YCR101C | 1 | 300825..301292,301294..302463,302478..303023 |
| III | YCL001W-A | 1or2 | 113074..113532,113549..113641,113644..114018 |
| III | YCL069W | 1or2 | 9427..9459,9463..11082 |
| IV | PRM7+YDL038C+YDL037C | 1or2 | 81982..382327,382330..384596,384600..385583 |
| IV | YDR134C | 1 | 721064..721474 |
| IV | YDR474C+YDR475C | 1or2 | 1407453..1409475,1409475..1409661,1409661..1410081 |
| IV | TTR1 | trimmed | 1471122..1471451 |
| V | YER066W | 1or2 | 289637..290797 |
| V | HVG1+YER039C-A | 1or2 | 228455..229480 |
| V | YEL077C+YEL076C+YEL075C | 1 | 264..4095,4097..4553,4553..5117,5134..5418,5420..5875 |
| V | YER189W+YRF1-2 | 1 | 571150..571463,571465..576520 |
| V | KHS1 | 1 | 565667..565792,565796..566398 |
| VI | AAD6+YFL057C | 1or2 | 14305..14919,14919..15431 |
| VI | BLM3+YFL006W | 1or2 | 123474..128885,128885..129904 |
| VI | YFL064C+65C+66C | 1 | 1..1516,1437..3008,3033..3338,3340..3846 |
| VI | YFR012W | 1or2 | 167880..168488,168487..169113,169116..169301 |
| VI | YFL042C+YFL043C | 1or2 | 45720..46157,46156..47745 |
| VIII | YHR218W+YHR219W | 1or2 | 557819..557854,557857..558606,558712..559820,559822..560167,560169..562043 |
| VIII | YHL049C+YHL050C | 1or2 | 445..2283,2285..3700,3728..4540 |
| VIII | YHR214W | 1or2 | 541503..542255,542259..543542 |
| IX | SDL1+YIL167W | 1or2 | 29032..29412,29416..30048 |
| IX | NIT1+YIL165C | 1or2 | 33718..34086,34090..34686 |
| IX | YIR043C+YIR044C | 1or2 | 437040..437990,437994..438176 |
| IX | YIL177C | 1 | 483..4988,4987..6147 |
| IX | HXT12 | 2 | 19515..19805,19808..21220 |
| X | PRM10+YJL107C | 1or2 | 217402..218554,218554..219713 |
| XI | PKT1+YKT9 | 1or2 | 68270..69079,69081..70220 |
| XI | YKL002W | new splice | 437416..437475,437544..438182 |
| XI | YKL033W-A | 1or2 | 74144..374305,374308..374853 |
| XII | AQY2+YLL053C | 1or2 | 35502..36044,36043..36360 |
| XII | YRF1-4+YLR462W+YLR464W | 1 | 1065951..1066556,1066567..1067079,1067082..1071230 |
| XII | SDC25+YLL017W | 2 | 112233..112502,112504..115992 |
| XIII | YMR084W+YMR085W | 1or2 | 436627..437412,437415..438788 |
| XV | YOL162W+YOL163W | 1or2 | 9596..10102,10106..10765 |
| XV | VPS5 | extended | 453769..453801,453804..455795 |
| XV | YOL048C | new splice | 240202..240945,241024..241308 |
| XV | ABP140 | 2 | 784855..785685,785687..786742 |
| XVI | YPL275W+YPL276W | 1or2 | 17948..18382,18386..18416,18418..19079 |
| XVI | YPL277C+YPL278C | 1or2 | 15053..15492,15494..16868 |

1 Pseudogene

2 Possible sequencing error (frameshift or stopcodon) or real mutation

(Bairoch and Apweiler, 1999) but may correspond to mutations in the sequenced strain. However, fragments pertaining to the same sequence should be represented as a single feature in the public databases. In addition to increased homology, data from YPD indicates identical phenotypes and expression patterns for some of these proposed merges. For example, *PRM7+YDL038+YDL037* have the same transcript profile (repressed by methylmethanesulphonate). Some of these proposed

Copyright © 2001 John Wiley & Sons, Ltd. 

gene extensions make previously predicted ORFs spurious.

### Disregarded (spurious) ORFs

Using the criteria described in Methods, 370 ORFs were disregarded (Table 2. and see **http://www.sanger.ac.uk/Projects/S_cerevisiae/spurious.shtml**). In agreement with Blandin *et al.* (2000), the ORFs which correspond to SAGE tags within LTRs have been reclassified as spurious.

### Orphans—very hypothetical

The discrimination between *S. cerevisiae* very hypothetical proteins and orphans which are more likely to be coding suggests 193 *S. cerevisiae* CDS should be described as *very hypothetical* ORFs (after the removal of ORFs which should be disregarded). Of these, 72 exhibit an overlap with another CDS (Table 3 and see **http://www.sanger.ac.uk/Projects/S_cerevisiae/veryhypothet.shtml**).

The G+C content, range and average was calculated for the fully partitioned ORFs on chromosomes I–V. ORFs were partitioned as: Real (characterised or well-conserved) = R; Sequence Orphans (possibly coding) = O; and Very Hypothetical (unlikely coding) = V. The mean G+C content for the partitioned ORF sets R:O:V are 40.24 : 40.37 : 38.84 respectively, which indicates there may be compositional differences between them. Even though the range of G+C for the very hypothetical proteins is smaller than for real (23.29 *vs.* 27.07), the sample standard deviation is greater (V = 5.06; R = 3.47).

### Discussion

### Novel genes

The three novel genes predicted by this analysis have now been incorporated into the MIPS database (M. Muensterkoetter, MIPS, pers comm).

Blandin *et al.* predicted 49 additional novel genes using interspecies sequence conservation, but some of these proposed new genes are spurious and others could be labelled *very hypothetical* using the criteria outlined in Methods. Some of these are predicted due to other non-CDS features. Others are extensions to existing genes. For example, *YMR013wa* is overlapped completely by a cellular RNA, *YGL258w* is part of *VPS5*, and *YER039ca* is part of *HGV1*. Other gene predictions from this dataset extend beyond the newly proposed coding region, and may correspond to regulatory regions, or to as yet undiscovered cellular RNAs. For example, *YDL159wa* is predicted to code for a 43 amino acid peptide (129 base pairs corresponding to the largest ORF) but the region of high similarity extends over 391 base pairs. Some predictions are derived from translations between 28 and 99 amino acids in length, and correspond to low complexity DNA sequence, often with only one species homologue. There are attendant risks in defining a CDS solely from an ORF and a statistically significant BLAST score (particularly with closely related organisms), as this may not always be biologically significant, or may pertain to a non-CDS feature. These predicted ORFs have been added to the Sanger annotation as miscellaneous features and will require further analysis before inclusion in the protein set.

### Merged and extended genes

Of the 46 alterations we propose, eight belong to subtelomeric duplicated elements and are possibly pseudogenes. For the remainder, those sequences not already corrected or confirmed or corrected by the sequencing of the genomic DNA will require resequencing for verification. However, frameshifts may still persist in the sequenced strain due to mutations.

### Disregarded spurious ORFs

Of the 370 genes proposed here to be *disregarded* ORFs, 227 were also predicted as unlikely to be coding by Zhang and Wang (2000). However, the Zhang and Wang analysis did not adequately differentiate between coding and non-coding sequences when applied to ORFs which were not in the *questionable* category of the MIPS database. Here, 18 of the 46 ORFs predicted to be non-coding for chromosomes I and II are now either functionally characterised (YPD) or conserved in distantly related organisms.

Malpertuy *et al.* (2000) propose that 91 of the ORFs annotated by MIPS as *questionable* (because they largely overlap other features) are actually real, based on similarity to the recently sequenced hemiascomycetes. We propose all of these ORFs should be *disregarded* as they will generate apparently

Copyright © 2001 John Wiley & Sons, Ltd.

Table 2. Spurious ORFs within other CDS or sequence features

| Spurious ORF | Overlapping feature | Spurious ORF | Overlapping feature | Spurious ORF | Overlapping feature |
|---|---|---|---|---|---|
| YAL004W | SSA1 | YDL034W | GPR1 | YER119C-A | SCS2 |
| YAL035C-A | MTW1 | YDL041W | SIR2 | YER138W-A | LTR |
| YAL043C-A | ERV46 | YDL050C | LHP1 | YER181C | LTR |
| YAL045C | new UVI31 | YDL062W | YDL063C | YFL013W-A | YFR055W |
| YAL058C-A | CNE2 | YDL068W | CBS1 | YFL067W | YFL013C |
| YAL064W-B | YAL065C | YDL094C | PMT5 | YFL068W | YFL066C |
| YAR009C | ty fragment | YDL096C | PMT1 | YFR024C | YFL066C |
| YAR010C | ty fragment | YDL118W | YDL119C | YFR056C | YFR024C-A |
| YBL012C | SCT1 | YDL151C | RPC53 | YGL024W | PGD1 |
| YBL053W | SAS3 | YDL152W | SAS10 | YGL042C | DST1 |
| YBL062W | SKT5 | YDL158C | STE7 | YGL069C | YGL068W |
| YBL065W | SEF1 | YDL163W | CDC9 | YGL072C | HSF1 |
| YBL070C | AST1 | YDL187C | YDL186W | YGL074C | HSF1 |
| YBL073W | AAR2 | YDL221W | CDC13 | YGL102C | RPL28 |
| YBL077W | ILS1 | YDL228C | SSB1 | YGL109W | YGL108C |
| YBL083C | RHK1 | YDR008C | TRP1 | YGL132W | YGL131C |
| YBL094C | YBL095W | YDR034C-A | LTR | YGL152C | CUP2 PEX14 |
| YBL096C | YBL095W | YDR048C | YDR049W | YGL165C | CUP2 |
| YBL100C | ATP1 | YDR053W | DBF4 | YGL168W | PMR1 |
| YBL107W-A | LTR | YDR094W | YDR093W | YGL199C | YGL198W |
| YBR051W | REG2 | YDR112W | YDR111C | YGL214W | SKI8 |
| YBR064W | YBR063C | YDR133C | YDR134C | YGL217C | KIP3 |
| YBR089W | POL30 | YDR149C | NUM1 | YGL218W | YGL219c |
| YBR090C | NHP6B | YDR154C | CHP1 | YGL235W | MTO1 |
| YBR099C | MMS4 | YDR187C | CCT6 | YGL239C | CSE1 |
| YBR113W | CYC8 | YDR193W | NUP42 | YGR011W | YGR010W |
| YBR116C | TKL2 | YDR199W | YDR200C | YGR018C | YGR017W |
| YBR124W | TFC1 | YDR203W | RAV2 | YGR022C | MLT1 |
| YBR174C | YBR175W | YDR230W | COX20 | YGR064W | SPT4 |
| YBR206W | KTR3 | YDR241W | LTR (29.16GC) | YGR073C | SMD1 |
| YBR219C | YBR220C | YDR269C | CCC2 | YGR114C | SPT6 |
| YBR224W | YBR223C | YDR271C | CCC2 | YGR115C | SPT6 |
| YBR226C | YBR225W | YDR278C | TRNA | YGR122C-A | LTR |
| YBR232C | PBP2 | YDR290W | RTT103 | YGR137W | YGR136W |
| YBR266C | YBR267W | YDR327W | SKP1 | YGR151C | RSR1 |
| YBR277C | DPB3 | YDR340W | TRNA and LTR | YGR160W | NSR1 |
| YCL022C | KCC4 | YDR355C | NUF1 | YGR164W | TRNA |
| YCL023C | KCC4 | YDR360W | VID21 | YGR176W | ATF2 |
| YCL041C | PDI1 | YDR366C | LTR | YGR190C | HIP1 |
| YCL042W | GLK1 | YDR396W | NCB2 | YGR219W | MRPI9 |
| YCL046W | YCL045C | YDR401W | DIT2 | YGR226C | AMA1 |
| YCL074W | ty fragment | YDR413C | YDR412W | YGR228W | SMI1 |
| YCL075W | ty fragment | YDR417C | RPL12B | YGR242W | YAP1802 |
| YCL076W | ty fragment | YDR426C | YDR425W | YGR259C | TNA1 |
| YCR013C | PGK1 | YDR431W | YDR430C | YGR265W | MES1 |
| YCR018C-A | LTR | YDR433W | NPL3 | YGR290W | MAL11 |
| YCR041W | MATALPHA1 | YDR442W | SSN2 | YHR125W | LTR |
| YCR049C | ARE1 | YDR445C | YDR444W | YHR145C | LTR |
| YCR050C | ARE1 | YDR455C | NHX1 | YHR214W-A | YHR214 |
| YCR064C | YCR063W | YDR467C | YDR466W | YIL060W | delta LTR |
| YCR087W | YCR087C-A | YDR509W | YDR509C | YIL080W | YIL082W |
| YDL009C | YDL010W | YDR521W | YDR520C | YJL007C | tRNA AND LTR |
| YDL011C | YDL010W | YDR526C | YDR527W | YJL009W | CCT8 |
| YDL016C | CDC7 | YDR537C | PAD1 | YJL018W | YJL019W |
| YDL023C | GPD1 | YEL076C-A | YELO76C | YJL022W | PET130 |
| YDL026W | YDL027C | YEL076W-C | YEL077C | YJL032W | BET4 |
| YDL032W | YDL033C | YER066C-A | YER067W | YJL075C | NET1 |

Copyright © 2001 John Wiley & Sons, Ltd. 

Table 2. *Continued*

| Spurious ORF | Overlapping feature | Spurious ORF | Overlapping feature | Spurious ORF | Overlapping feature |
|---|---|---|---|---|---|
| YJL086C | YJL087C | YLR261C | YPT6 | YNL170W | PDS1 |
| YJL119C | YJL118W | YLR279W | YLR281C | YNL171C | APC1 |
| YJL142C | YAK1 | YLR280C | YLR281C | YNL174W | NOP13 |
| YJL152W | YJL151C | YLR282C | YLR283W | YNL184C | MRPL19 |
| YJL169W | SET2 | YLR317W | TAD3 | YNL203C | SPS19 |
| YJL175W | SWI3 | YLR322W | SFH1 | YNL205C | SPS18 |
| YJL188C | RPL39 | YLR331C | MID2 | YNL226W | YNL227C |
| YJL195C | CDC6 | YLR334C | LTR | YNL228W | YN227 |
| YJL202C | PRP21 | YLR338W | VRP1 | YNL235C | SIN4 |
| YJL211C | PEX2 | YLR339C | RPP0 | YNL266W | IST1 |
| YJL220W | FSP2 | YLR349W | DIC1 | YNL276C | MET2 |
| YJR018W | ESS1 | YLR358C | RSC2 | YNL285W | LTR and Trna |
| YJR020W | TES1 | YLR374C | STP1 | YNL296W | MON2 |
| YJR023C | LSM8 | YLR379W | SEC61 | YNL319W | HXT14 |
| YJR037W | HUL4 | YLR428C | CRN1 | YNL324W | FIG4 |
| YJR038C | YJR039w | YLR434C | YLR435W | YNR005C | VPS27 |
| YJR071W | YJR070C | YLR444C | ECM7 | YNR042W | COQ2 |
| YJR079W | YJR080C | YLR458W | NBP1 | YOL013W-A | LTR |
| YJR087W | YJR088C | YLR463C | YRF1-4 | YOL035C | YOL036W |
| YJR128W | ZMS2 | YLR465C | YRF1-4 | YOL037C | YOL036W |
| YKL030W | MAE1 | YML010C-B | SPT5 | YOL046C | YOL045W |
| YKL036C | YKL037W UGP1 | YML010W-A | SPT5 | YOL050C | GAL11 |
| YKL053W | YKL052C | YML013C-A | YML013W | YOL079W | REX4 |
| YKL076C | YKL075C | YML035C-A | SRC2 | YOL099C | PKH2 |
| YKL083W | YKL082C | YML048W-A | PRM6 | YOL106W | LTR |
| YKL111C | ABF1 | YML058C-A | CMP2 | YOL134C | HRT1 |
| YKL118W | VPH2 | YML095C-A | GIM5 | YOL150C | GRE2 |
| YKL123W | SSH4 | YML100W-A | ARG81 | YOR053W | YOR054C |
| YKL131W | RMA1 | YML102C-A | CAC2 | YOR055W | YOR054C |
| YKL136W | ALP2 | YML117W-A | YML117W | YOR068C | new VPS5 |
| YKL147C | YKL146W | YMR046W-A | LTR | YOR082C | YOR083W |
| YKL153W | GPM1 | YMR052C-A | FAR3 and STB2 | YOR102W | OST2 |
| YKL169C | MRPL38 | YMR075C-A | YMR075W | YOR105W | YOR104W |
| YKL177W | STE3 | YMR119W-A | YMR119W | YOR121C | GCY1 |
| YKL202W | MNN4 | YMR135W-A | YMR135C | YOR135C | IDH2 |
| YKR012C | PYR2 | YMR153C-A | NUP53 | YOR139C | SFL1 |
| YKR033C | DAL80 | YMR158C-B | LTR | YOR146W | YOR145C |
| YKR035C | FTI1 | YMR158W-A | APG16 | YOR169C | GLN4 |
| YKR047W | NAP1 | YMR172C-A | HOT1 | YOR170W | LCB4 |
| YLL020C | KNS1 | YMR173W-A | DDR48 | YOR200W | PET56 |
| YLL037W | PRP19 | YMR193C-A | YMR194W | YOR203W | HIS3 and DED1 |
| YLL044W | RPL8B | YMR244C-A | YMR245W | YOR218C | RFC1 |
| YLL047W | RNP1 | YMR290W-A | HAS1 | YOR225W | ISU2 |
| YLR041W | YLR040C | YMR294W-A | YMR295C | YOR248W | SRL1 |
| YLR062C | RPL22-A | YMR306C-A | FKS3 | YOR263C | YOR264W |
| YLR076C | RPL10 | YMR316C-A | DIA1 | YOR277C | CAF20 |
| YLR101C | ERG27 | YMR316C-B | YMR317W | YOR282W | PLP2 |
| YLR140W | RRN5 | YNL013C | YNL014W | YOR300W | BUD7 |
| YLR169W | APS1 | YNL017C | Trna | YOR309C | NOP58 |
| YLR171W | APS1 | YNL043C | YIP3 | YOR325W | YOR324C |
| YLR198C | SIK1 | YNL057W | YNL058C | YOR331C | VMA4 |
| YLR202C | YLR201C | YNL089C | RHO2 | YOR333C | MRS2 |
| YLR217W | CPR6 | YNL105W | INP52 | YOR345C | REV1 |
| YLR230W | CDC42 | YNL109W | YNL108C | YOR364W | YOR365C |
| YLR232W | YLR231C | YNL114C | RPC19 | YOR366W | YOR365C |
| YLR235C | TOP3 | YNL120C | YNL119W | YOR379C | YOR378W |
| YLR252W | YLR251W | YNL140C | RLR1 | YPL035C | YPL034W |

Copyright © 2001 John Wiley & Sons, Ltd.

Table 2. *Continued*

| Spurious ORF | Overlapping feature | Spurious ORF | Overlapping feature | Spurious ORF | Overlapping feature |
|---|---|---|---|---|---|
| YPL044C | NOP4 | YPL251W | YAH1 | YPR087W | SRP54 |
| YPL073C | UBP16 | YPR002C-A | LTR | YPR099C | YPR0100W |
| YPL102C | YPL101W | YPR038W | YPR037C | YPR123C | CTR1 |
| YPL114W | YPL113C | YPR039W | YPR037C | YPR126C | YPR125W |
| YPL136W | YPL137W | YPR044C | RPL43A | YPR130C | SCD6 |
| YPL142C | RPL33A | YPR050C | MAK3 | YPR136C | RRP9 |
| YPL182C | YPL181W | YPR053C | NHP6A | YPR177C | PRP4 |
| YPL185W | YPL186c | YPR059C | YMC1 | YPR197C | SGE1 |
| YPL197C | RPL7B | YPR076W | OPY2 | | |
| YPL238C | SUI3 | YPR077C | YPR078C | | |

significant, but spurious, TBLASTX matches to alternative frames of the real gene (anti-sense or sense-different reading frame).

Of the ORFs previously defined as questionable but now proposed to be coding by Malpertuy *et al.* (2000), and retrievable from the Genolevures website (**http://cbi.labri.u-bordeaux.fr/Genolevures/Genolevures.php3**), at least 107 out of 136 occur in overlapping pairs. These pairs have two significant TBLASTX hits when the ascomycete DNA is compared to the *S. cerevisiae predicted* protein set; the best score belonging to the real coding sequence and a lower score generated by the overlap with the spurious ORF and an alternative translation of the closely related organism's DNA. This is illustrated by the three pairs of overlapping genes *YGR220C*/*YGR219w*, *YOR054c*/*YOR055w*, and *YDR443C*/*YDR442w* in Table 4 (data from the Genolevures website). The correct reading frame should also be apparent if levels of synonymous and nonsynonymous nucleotide substitution are calculated for the aligned regions.

After the merging of sequences identified in *Merged and extended genes* (Table 1), only eight genes of known or inferred function in the entire *S. cerevisiae* genome remain overlapping. The overlaps and their orientations are listed in Table 5. The longest overlaps observed were 55 and 34 amino acids, which are possibly attributable to sequencing anomalies, or deletions; the other six are 10 amino acids or less, and predominantly C-terminal.

Overlapping CDS features are also rare in *Sz. pombe*. Of 4189 genes which are characterised or conserved, only three pairs have an overlap greater than 10 amino acids in length, none of which were at the N terminus. Moreover, since the completion of the *S. cerevisiae* genome, no function or biologically significant similarity to any other sequenced organism has been observed for any of the largely overlapping ORFs designated here as spurious. This is despite the major efforts of EUROFAN and other functional genomics studies to determine the function of every yeast gene, and the exponential increase in protein sequences deposited in the public databases.

Considering the rarity of overlapping genes in both yeasts, and the absence of unequivocal functional evidence in support of the coding integrity of any of the spurious ORFs which are wholly or largely overlapping real genes, the likelihood that any encode for proteins is minimal. Therefore, it would be prudent to remove them completely from the genome totals and label them accordingly in the public databases.

### Very hypothetical proteins

One advantage of discriminating between sequence orphans likely to be coding, and *very hypothetical* orphans, is that these regions of DNA can be easily partitioned as a subset, facilitating the identification of other features by bioinformatics analyses.

Despite over 1000 *Sz. pombe* experimental gene characterisations, only three of the 177 *Sz. pombe* ORFs annotated as '*very hypothetical protein*' have so far been shown to be protein coding. One of these, git11, is a 76 amino acid protein, and is below the threshold imposed on length, but appeared to have coding potential. The size distribution and splicing frequency of *Sz. pombe* orphans, when compared to genes of known or inferred function, suggests that a larger proportion may not be real (manuscript in preparation).

Copyright © 2001 John Wiley & Sons, Ltd. 

Table 3. Very hypothetical proteins with no homology and low coding potential

| CDS | CDS | CDS | CDS |
|---|---|---|---|
| YAL069W | YEL045C | YIL100W | YLR385C |
| YAL066W | YEL033W | YIL086C | YLR400W |
| YAR029W | YEL010W | YIL054W | YLR402W |
| YAR030C | YER053C-A | YIL025C | YLR415C |
| YAR047C | YER066C-A | YIL012W | YLR416C |
| YAR053W | YER071C | YIL059C | YLR184W |
| YAR060C | YER084W | YIL058W | YML108W |
| YAR064W | YER091C-A | YIL032W | YML090W |
| YAR069C | YER092W | YIR020C | YML089C |
| YAR070C | YER093C-A | YIR020W-B | YML084W |
| YBL071C | YER097W | YIR044C | YMR031W-A |
| YBL048W | YER121W | YJL199C | YMR151W |
| YBL129C-A | YER181C | YJL182C | YMR057C |
| YBR012C | YER187W | YJL150W | YMR086C-A |
| YBR013C | YER189W | YJL135W | YMR194C-A |
| YBR027C | YFL015C | YJL120W | YMR245C |
| YBR032W | YFL019C | YJL027C | YMR304C-A |
| YBR209W | YGL261C | YJL015C OR YJL016W | YMR320W |
| YBR300C | YGL260W | YJR114W OR YJR113C | YMR324C |
| YBR134W | YGL204C | YJR146W | YNL338W |
| YBR178W | YGL193C | YJL067W OR YJL066C | YNL337W |
| YBR190W | YGL188C | YJL065C OR YJL064W | YNL303W |
| YCL021W-A | YGL182C | YJL062W-A | YNL300W |
| YCR006C | YGL177W | YJL052-A | YNL226W |
| YCR022C | YGL149W | YKL162C-A | YNL198C |
| YCR025C | YGL052W | YKL102C | YNL179C |
| YCR043C | YGL051W | YKL044W | YNL149C OR YNL150W |
| YCR085W | YGL041C | YKL031W | YNL146W |
| YCR102W-A | YGR025W | YKL115C | YNL028W |
| YDL242W | YGR050C | YKR032W | YNR025C OR YNR024W |
| YDL196W | YGR051C | YKR041W OR YKR040C | YNR001W-A |
| YDL172C OR YDL173W | YGR069W | YKR073C | YOL166C |
| YDL162C | YGR139W | YKL106C-A | YOL026C |
| YDL157C | YGR182C | YKL018C-A | YOR012W OR YOR013W |
| YDR114C | YGR269W | YLL030C | YOR024W |
| YDR029W | YGR291C | YLR111W | YOR041C OR YOR042W |
| YDR157W | YGR293C | YLR112W | YOR225W |
| YDR209C | YGR161W-A | YLR123C OR YLR122C | YOR235W |
| YDR220C | YGR271C-A | YLR161W | YOR268C |
| YDR320C-A | YHL045W | YLR162W | YOR304C-A |
| YDR504C | YHL005C | YLR255C | YOR343C |
| YDR491C | YHR217C | YLR269C | YPL261C |
| YDR034W-B | YHR130C | YLR294C | YPL205C |
| YDR070C | YHR139C-A | YLR311C | YPR012W |
| YEL074W | YHR173C | YLR302C | YPR092W |
| YEL073C | YIL174W | YLR346C | YPR142C OR YPR143W |
| YEL068C | YIL163C | YLR365W | YPR146C |
| YEL059W | YIL141W | YLR366W | YPR150W OR YPR151C |
| YPR074W-A | | | |

### Implications for post genomics

Many of the spurious overlapping ORFs included in the public databases, and proposed as disregarded ORFs by this analysis, have associated functional genomics data which could be artefacts. The original yeast microarrays (using PCR products), were not strand specific with respect to the probes (DeRisi *et al.*, 1997), and opposite strand transcripts could hybridise to these array spots (D. Vetrie, perscomm).

Copyright © 2001 John Wiley & Sons, Ltd.

Table 4. Real/false pairs

| Ascomycete DNA | Real gene | Length | P value | Blast Score | Disregarded ORF | Length | P value | Blast Score | Overlap Length |
|---|---|---|---|---|---|---|---|---|---|
| AR0AA003F06TP1 | YGR220C MRPL9 | 269 | 7e-69 | 72 | YGR219w | 113 | 0.01 | 31 | 100 |
| AR0AA005F12TP1 | YOR054c | 674 | 2e-09 | 52 | YOR055w | 144 | 2e-10 | 49 | 127 |
| AR0AA008C07CP1 | YDR443C SSN2 | 1420 | 2e-16 | 130 | YDR442w | 130 | 3e-12 | 66 | 120 |

Positive signals may also result from overlapping UTRs. Not unexpectedly, many of the disregarded ORFs have transcript profiles similar to the overlapping characterised gene. Gene knockouts of spurious ORFs may give phenotypes, particularly if they affect overlapping strand ORFs, promoters, or other cellular RNAs. It has been observed that some of the knockouts of overlapping ORFs have essentially the same, or similar phenotype to the real adjacent gene. These transcript and phenotype artefacts, attached to the database entries, lend these predictions false credibility as proteins. The inclusion of spurious ORFs may therefore affect the accuracy of any previous global analysis of transcription or redundancy.

### New gene number estimate

Our analysis provided a new estimate of gene number for each *S. cerevisiae* chromosome. These are provided in Table 6.

When *S. cerevisiae* was first published, 6275 ORFs were predicted; 390 of these were proposed to be spurious giving a probable gene number of 5885 (Goffeau *et al.*, 1996). The data used for our analysis (SGD) consisted of 6282 ORFs, of which 370 have been disregarded, giving a new *maximum* upper limit of 5804. The removal of 42 pseudo or frame-shifted sequences, and 193 very hypothetical proteins further reduces this total to 5570. This is likely to be closer to the true upper limit, because the criteria used for the determination of very hypothetical proteins are quite conservative. There is a possibility that a small number of the very hypothetical proteins may eventually be determined to be coding, but size distribution (unpublished) indicates that we may still be over estimating the number of small ORFs.

Table 5. Real *S. cerevisiae* genes with overlaps

| Gene 1 | Gene 2 | Overlap (AA) | Orientation |
|---|---|---|---|
| BUD5 | MATALPHA2 | 0.3 | N/C |
| AUA1 | YFL010C | 55 | C/C |
| AMD1 | PRP38 | 1 | C/C |
| ECM12 | YHR022C | 34 | C/C |
| VPS38 | YLR361C | 10 | C/C |
| YML096W | RAD10 | 1 | C/C |
| YNL246W | YNL245C | 0.3 | C/C |
| CTF19 | YPL017C | 8 | C/C |

N = N terminus, C = C terminus, AA = Amino acid length.

Malpertuy *et al.* predicted a gene number of 5651. In addition, using two different statistical methods, they estimated that the actual number of protein coding ORFs should be either 5542 or 5552, but do not account for the differences between their predicted number of 5651 and the statistical calculations. The statistical calculations are closer to the number of genes predicted by our analysis (5570 or fewer). The discrepancies could be due to the inclusion of novel genes which are in fact spurious (see Discussion, Novel Genes), or the inclusion of genes previously defined as questionable, but proposed by this analysis to be disregarded (see Discussion, Spurious ORFs).

### What are the remaining orphans?

Data obtained by Gaillardin *et al.* (2000) demonstrated that ascomycete specific genes are highly represented in the functional classes of *cell wall organisation*, *extracellular/secreted proteins*, and *transcriptional regulators* suggesting that they diverge more rapidly than other classes of genes. In *Sz. pombe*, many remaining orphans are low complexity or repetitive proteins e.g. serine-rich with low similarity to alpha-agglutins and other cell surface proteins, or proteins with basic charged regions which may correspond to transcription factors. It may be that most orphans correspond to genes which have diverged so much that they are unrecognisable, rather than novel genes. It is therefore possible that the majority of orphans are genes which have diverged more rapidly and that the number of truly species specific genes is very small.

A comparison of the refined orphan sets of

Copyright © 2001 John Wiley & Sons, Ltd.

Table 6. Predicted *S.cerevisiae* gene numbers, by chromosome

| Chromosome number | I | II | III | IV | V | VI | VII | VIII | IX | X | XI | XII | XIII | XIV | XV | XVI | Total |
|---|---|---|---|---|---|---|---|---|---|---|---|---|---|---|---|---|---|
| ORF No. (Goffeau *et al.* 1996) | 110 | 422 | 172 | 812 | 291 | 135 | 572 | 288 | 231 | 387 | 334 | 547 | 487 | 421 | 569 | 497 | 6275 |
| Questionable (Goffeau *et al.* 1996) | 3 | 30 | 12 | 65 | 13 | 5 | 57 | 12 | 11 | 29 | 20 | 41 | 30 | 23 | 3 | 36 | 390 |
| SGD 2000 ORFs | 107 | 428 | 173 | 819 | 288 | 134 | 571 | 284 | 224 | 387 | 335 | 547 | 491 | 421 | 573 | 500 | 6282 |
| Spurious non coding | 8 | 27 | 16 | 61 | 7 | 3 | 39 | 3 | 2 | 25 | 19 | 38 | 27 | 27 | 38 | 28 | 368 |
| Sanger new | 98 | 397 | 154 | 743 | 275 | 122 | 525 | 275 | 215 | 354 | 317 | 497 | 455 | 391 | 527 | 459 | **5804** |
| Pseudo or frameshift | 2 | 2 | 3 | 1 | 2 | 2 | 2 | 3 | 5 | 5 | 2 | 4 | 3 | 1 | 3 | 2 | 42 |
| Sanger very hypothetical | 10 | 13 | 6 | 16 | 16 | 1 | 21 | 7 | 12 | 14 | 11 | 19 | 12 | 13 | 11 | 10 | 192 |
| Final | 86 | 382 | 145 | 726 | 257 | 119 | 502 | 265 | 198 | 335 | 304 | 474 | 440 | 377 | 513 | 447 | **5570** |

*Sz. pombe* and *S. cerevisiae* will aid the detection of subtle homologies and physical similarities between the sequence orphans themselves, or orphans and previously characterised genes. For example, the final subunit of *Sz. pombe* RNA polymerase III (the homologue of *S. cerevisiae* RPC31) was identified due to similarity in amino acid length and the presence of an acidic C terminus, despite a low similarity score (Richard Maraia, NICHD, NIH, Bethesda and George Shpakovski, Russian Academy of Sciences, Moscow. pers comm).

Global comparison of the remaining orphans will facilitate definition of the sets of genes necessary for unicellular eukaryotic life. However, to do this effectively, it is important that a distinction is first made between orphans and spurious ORFs (Malpertuy *et al.*, 2000).

### Conclusions

A substantial proportion of small orphans are probably not protein coding, yet may define other genome features (regulatory regions, cellular RNAs or even gene-free regions which may be involved in higher order chromosome structure). These may contain spurious ORFs which, if defined as CDS, appear to generate matches at the protein level. Spurious gene predictions, with associated artefactual functional genomics data, will exclude these regions of DNA from being inspected for non-CDS features. Attaching a suitable annotation to these would facilitate the detection of authentic features.

It is important to differentiate firstly between orphans and disregarded spurious ORFs, and secondly, between likely real orphans and very hypothetical orphans. Refinement of the orphan sets of sequenced genomes will enable the detection of more subtle homologies and other physical similarities between the real orphans.

As the number of orphans is gradually eroded by the removal of non-coding ORFs and the detection of distant homologues, it will become easier to determine how many truly species specific genes exist in the *Sz. pombe* and *S. cerevisiae* genomes.

The annotation of an ORF's status within the public datasets is important for both functional genomics and bioinformatics. The costs of reagents, labour and curation of 370 ORFs which should be disregarded in functional genomics analyses are not trivial, they account for roughly 5% of the total effort. Bioinformatics on proteome data to examine amino acid composition, charge, etc. require accurate datasets (perhaps with different confidence levels attributed). Integration of contextual information on a gene-by-gene basis to determine status will enable the targeting of future research toward genes which are more likely to be coding.

As more analyses are performed, we should get closer to the absolute gene number. Taken in combination, previous analyses and the interpretion of the biological context of the ORF should enable better estimates of probable gene and orphan number for this yeast.

### Data availability

Updated EMBL format sequences (containing nearly 12000 annotations) which can be examined in Artemis and a one-gene one-protein FASTA format protein translations database are available from the Sanger Centre ftp site (ftp://ftp/pub/yeast/SCreannotation).

The EMBL entries will continue to be maintained (and will be resubmitted to EMBL with permission

Copyright © 2001 John Wiley & Sons, Ltd.

of the original authors). Further refinement of the datasets described will include:

1. New similarity information from BLASTX.
2. EST/cDNA mappings.
3. Regulatory region identification/mapping.
4. Inclusion of other annotated features (keys and qualifiers) from individual GenBank/EMBL entries.

### References

Altschul SF, Gish W, Miller W, Myers EW, Lipman DJ. 1990. Basic Local Alignment Search Tool. *J Mol Biol* **215**: 403–410.

Bairoch PBA. 1994. A generalized profile syntax for biomolecular sequences motifs and its function in automatic sequence interpretation. In *ISMB-94; Proceedings 2nd International Conference on Intelligent Systems for Molecular Biology*. AAAIPress; 53–61.

Bairoch PBA, Apweiler R. 1999. The SWISS-PROT protein sequence data bank and its supplement TrEMBL in 1999. *Nucleic Acid Res* **27**(1): 49–54.

Bateman A, Birney E, Durbin R, Eddy S, Finn R, Sonnhammer E. 1999. Pfam 3.1: 1313 multiple alignments and profiles HMMs match the majority of proteins. *Nucleic Acid Res* **27**(1): 260–262.

Berbee ML, Taylor JW. 1993. Dating the evolutionary radiations of the true fungi. *Can J Bot* **71**(1114): 1127.

Birney E, Thompson J, Gibson T. 1996. PairWise and SearchWise: finding the optimal alignment in a simultaneous comparison of a protein profile against all DNA translation frames. *Nucleic Acid Res* **24**(14): 2730–2739.

Blandin G, Durrens P, *et al*. 2000. The genome of *Saccharomyces cerevisiae* revisited. *FEBS Lett* 1–6.

DeRisi JL, Vishwanath R, Brown P. 1997. Exploring the metabolic and genetic control of gene expression on a genomic scale. *Science* **278**: 681–686.

Dujon B. 1996. The yeast genome project: what did we learn? *Trends Genet* **12**(7): 263–270.

Gaillardin C, Duchateau-Nguyen G, Tekaia F, *et al*. 2000. Genomic exploration of the hemiascomycetous yeasts: 21. Comparative functional classification of genes. *FEBS Lett* 134–149.

Goffeau A, Barrell B, Bussey H, *et al*. 1996. Life with 6000 genes. *Science* **274**: 546–567.

Hieter P, Boguski M. 1997. Functional genomics: it's all how you read it. *Science* **278**: 601–602.

Lowe T, Eddy S. 1997. tRNAscan-SE: a program for improved detection of transfer RNA genes in genomic sequence. *Nucleic Acid Res* **25**: 955.

Mackiewicz P, Kowalczuk M, Gierlik A, Dudek MR, Cebrat S. 1999. Origin and properties of non-coding ORFs in the yeast genome. *Nucleic Acid Res* **27**: 3503–3509.

Malpertuy A, Tekaia F, Casaregola S, *et al*. 2000. Genomic exploration of the hemiascomycetous yeasts: 19. Ascomycetes-specific genes. *FEBS Lett* 113–121.

Mewes H, Albermann K, Bahr M, *et al*. 1997. Overview of the yeast genome. *Nature 387. supplement.*

Oliver S. 1996. From DNA sequence to biological function. *Nature* **379**: 597–600.

Oliver SG, van der Aart QJM, Agostoni-Carbone ML, *et al*. 1992. The complete DNA sequence of yeast chromosome III. *Nature* **357**: 38–46.

Pearson W, Lipman D. 1988. Improved tools for biological sequence comparison. *Proc Natl Acad Sci* **85**: 2444–2448.

Rutherford K, Parkhill J, Crook J, Horsnell T, Rice P, Rajandream M, Barrell B. 2000. Artemis: sequence visualization and annotation. *Bioinformatics* **16**(10): 944–945.

Sharp P, Cowe E. 1991. Synonymous codon usage in *Saccharomyces cerevisiae*. *Yeast* **7**: 657–678.

Sonnhammer E, Durbin R. 1994. A workbench for large scale sequence homology analysis. *Comput Appl Biosci* **10**: 301–307.

Stoesser G, Tuli M, Lopez R, Sterk P. 1999. The EMBL Nucleotide Sequence Database. *Nucleic Acid Res* **27**(1): 18–24.

Xiang Z, Moore K, Wood V, *et al*. 2000. Analysis of 114 kb of DNA sequence from fission yeast chromosome 2 immediately centromere-distal to *his 5*. *Yeast* **16**: 1405–1411.

Zhang C-T, Wang J. 2000. Recognition of protein coding genes in the yeast genome at better than 95% accuracy based on the Z curve. *Nucleic Acid Res* **28**: 2804–2814.

Copyright © 2001 John Wiley & Sons, Ltd. 
